# Conformity behavior in crises: evidence from the COVID-19 epidemic in China

**DOI:** 10.3389/fpsyg.2024.1428075

**Published:** 2024-06-28

**Authors:** Yujiao Yao, Shanshan Liu, Gaoyu Chen, Yang Yang, Jiaxin Yang

**Affiliations:** ^1^School of International Studies, Sichuan University, Chengdu, China; ^2^School of Business Administration, Faculty of Business Administration, Southwestern University of Finance and Economics, Chengdu, China; ^3^China Shipbuilding Trading Co., Ltd., Beijing, China; ^4^School of Mathematics, Southwestern University of Finance and Economics, Chengdu, China

**Keywords:** conformity behavior, COVID-19, mass health crisis, risk perception, social influence

## Abstract

Once a mass health crisis breaks out, it causes concern among whole societies. Thus, understanding the individual’s behavior in response to such events is key in government crisis management. From the perspective of social influence theory, this study adopts the empirical research method to collect data information in February 2020 through online survey, with a view to comprehensively describe the individuals’conformity behavior during the COVID-19 outbreak in China. The individual’s conformity behavior and new influencing factors were identified. The results revealed that affective risk perception, cognitive risk perception, and individual risk knowledge had a positive significant impact on normative influence. Affective risk perception and individual risk knowledge had a positive significant on informative influence. Cognitive risk perception did not significantly impact informative influence. Informative influence and normative influence had a positive effect on conformity behavior. These results have significant implications for the management behavior of the government.

## 1 Introduction

The Coronavirus Disease 2019 (COVID-19) global mass health crisis poses a large threat to survival, social order, and public security, and will also damage social life and economic stability (Toubes et al., 2021). Once a mass health crisis breaks out, it becomes the focus of the whole of society and directly challenges the government’s governing ability (Liu et al., 2021). In these times, the individual often experiences fear and tension coupled with the pressures of time and medical resources. Furthermore, people’s conformity behavior is particularly prominent when considering the management of mass health crises (Normandin and Therrien, 2016).

At the beginning of the COVID-19 outbreak in China, especially in February, various topics surrounding the epidemic became popular discussion points. The popularity of social media, such as Weibo and WeChat, and mobile information terminals, such as mobile phones, have long been widely used in China. Indeed, posting, obtaining, commenting, and forwarding information has become daily behavior in many citizens. Given that members of the public do not need to reveal their true identity, some rumors can appear on online networks, and these are forwarded frantically online (Chen et al., 2020). At the same time, information circulating on the internet will also affect conformity behavior of the general public (Garfin et al., 2020). Online conformity behavior and offline conformity purchase behavior that occurs during the prevention and control of major emergencies can cause some chaos and is not conducive to restoring normal order. Therefore, the government should pay close attention to the individual’s conformity behavior while striving toward the prevention and control public crisis.

Based on these issues, this study investigated conformity behavior during China’s COVID-19 mass health crisis. We adopted the social influence theory to analyze data collected online in February 2020, and risk perception (affective risk perception and cognitive risk perception) was included in the model. The main purpose of this study was to identify whether individual risk perception influences individual conformity behavior in the context of a mass health crisis. We believe that our results provide a reliable reference for government organizations to formulate public health risk management strategies.

## 2 Theory and hypotheses

### 2.1 Risk perception

In modern society, mass health crises have many new characteristics, such as uncertainty, and globalization. In addition, risk perception also depends on individual’s cognition, judgment, and decision making considering the objective risk. Risk perception is the individual’s beliefs, attitude, judgment, and emotion about potential danger (Sjöberg, 2000). The individual’s risk perception will affect behavior, whereby a stronger risk perception will be more likely to trigger the individual’s defensive decision-making behavior, and it can be affected by social, psychological, and cultural factors (Slovic, 2016). Therefore, the individual’s risk perception cannot be measured by the cognitive attributes of risk alone, but should also consider psychological experience. Risk perception and risk decision behaviors are the consequence of interactions between rationality and affective. Cognitive evaluations are also heavily influenced by specific affective and loss-based ethics according to individual risk assessment models. In daily life, people’s risk perceptions can be quite different. For example, experts often analyze risk details rationally, systematically, and objectively based on the factual basis of risk (such as mortality estimates and technical controllability of disasters); however, for the general public, the technology-statistics orientation and probability estimations do not contribute to risk perception. Rather, risk perception is based on personal emotions, and irrational, intuitive, and subjective “experience” risk attributes (such as threats to family members and the impact on daily life) (Böhm and Pfister, 2000). Applying dual processing theory in cognitive psychology, risk managers are beginning to realize how “affectivity” can act as an underlying mechanism that governs risk assessment, and which complements “cognition” (Peters and Slovic, 1996; Ferrer et al., 2016).

Affective risk perception is a type of heuristic information processing that is fast, intuitive, parallel, and spontaneous, in which cognitive resources have little involvement. Cognitive risk perception is slow, cautious, sequential, and controllable, and requires analytical information processing of cognitive resources. Some research has shown that negative emotions, such as anxiety, fear, anger, and psychological stress, contribute to a specific type of risk perception. In short, the individual both “thinks” and “feels” risks at the same time (Lerner et al., 2003; Sobkow et al., 2016). Scholars believe that there are different mechanisms underlying affective risk perception and cognitive risk perception. The former often has a stronger explanatory power for individual risk decision-making (Loewenstein et al., 2001; Lerner et al., 2003; Ng et al., 2018). The individual’s health care behavior in the face of cancer risk is reportedly more driven by affective risk perception than cognitive risk perception (Loewenstein et al., 2001). Some studies have shown that cognitive risk perception is more predictive of behavior in food safety risk situations (Ng et al., 2018). Risk perception often affects people’s actions; that is, a stronger risk perception is more likely to trigger defensive behaviors and decisions, and to be affected by the behavior of others. Decision science has shown that when facts are effectively communicated to the public through the media, a more accurate understanding of the risks is formed (Saltkjel et al., 2017; Ng et al., 2018).

To summarize, this study used the dual processing mechanism of risk perception into the research model, and examined the impact of two types of risk perception on people’s conformity behavior during the COVID-19 mass health crisis.

### 2.2 Individual risk knowledge

The individual risk knowledge means that individuals have knowledge about the hazards, transmission, protection, and national policies of COVID-19. The concept of individual risk knowledge comes from brand knowledge in marketing; this refers to the descriptive and evaluative information about a brand in the minds of consumers, and their understanding of cognition, emotions, and thoughts induced by the brand (Keller, 2003; Füller et al., 2008). During public crisis events, individual’s daily behavior focuses on reducing the uncertainty of behavioral results and avoiding personal losses. Ways to reduce decision-making risks include seeking advice from opinion leaders and searching for more extensive information. When individuals have little knowledge, their ability to search for external information is inadequate, and they lack the ability to judge the pros and cons of complex environmental information. To reduce the uncertainty of decision-making results, they seek more information from opinion leaders, which means that they are influenced by information (Dholakia, 1997).

Individuals who have more knowledge of mass health crisis risks not only have more understanding of the crisis itself, but also have a certain affective awareness of crisis. When individuals have enough risk knowledge, they can be considered as behavior experts. Namely, they are confident in the results of their behaviors and do not conduct extensive information searches; thus, they are less affected by information. We hypothesized that individual’s level of risk knowledge affects the degree to which they are influenced by normative and informative.

### 2.3 Social influence theory

Social influence refers to the idea that an individual’s beliefs, attitudes, emotions, and behavior will change under the influence of social forces, which include the environment, public opinion, and even fashion (Katona et al., 2011). The impact of social influence on conformity behavior can be divided into normative social influence and informative social influence (Katona et al., 2011). Normative social influence causes individuals to obey the expectations of Key Opinion Leader. Realizing social expectations can produce good feelings and a sense of belonging. Normative influence refers to the normative pressure brought by surrounding groups; individuals will try to reduce this normative pressure by adopting the same behavior as other group members (Katona et al., 2011; Iyengar et al., 2015).

Citizens can gain more information about public crises through informal conversations with friends and family and observation of other people’s behavior. The informative influence mainly manifests as (1) the public’s breadth of understanding of the current events, and (2) the informative influence generated in the conformity process, which can help consumers confirm the rationality of existing information and reduce the uncertainty of their own behavior, even if the individual does not obtain the latest or known information (Bearden et al., 1989; Du and Kamakura, 2011). Normative influence emphasize the consistency between affected people and influencers to maintain a closer social relationship, and show a social identity and behavior compliance (Iyengar et al., 2015). Informative influence emphasize the point that consumers treat others as an informational source (Bearden et al., 1989; Iyengar et al., 2015).

Informative social influence refers to accepting other people’s information as factual. In uncertain decision making, the impact of information on behavior can be varied. On the one hand, the informative influence can be realized by internalization. The decision-making process uses the informative influence as a kind of social signal, and combines this with personal signals to change the individual attitude by changing the belief judgment of the event. In this case, people accept the influence of information because the corresponding behavior is consistent with their own value system. The actions taken through internalization may be to maximize their own interests. On the other hand, informative influence can be heuristic and simplified to influence other variables. Heuristic simplification is a rapid response tool in humans to improve the speed and efficiency of decision making and reduce the cost of decision making in biological and social evolution. In a crisis environment, individuals are inevitably at a loss due to panic and fear. Therefore, it is natural to observe and imitate others’ actions (Burnkrant and Cousineau, 1975; Abrams and Hogg, 1990; Cialdini and Goldstein, 2004). Some researchers have reported that attitude is a multiple structure, and the objective reflection of all stimuli is determined by the attitude of the object (Park, 2000). In the absence of information, that is, insufficient knowledge as a result of either information being unknown to officials or because information that is not communicated effectively, ambiguity will lead to a high degree of threat assessment. This happened during the H1N1 crisis; when uncertainty increased, the sense of uncontrollability increased and anxiety intensified, which led to conformity behavior. This is also expected to occur in the context of COVID-19 (Chen et al., 2020).

In summary, the context of health crises is not taken into consideration when previous studies discussed the influence of risk perception’s dual operational mechanism on individual behaviors. Besides, scholars have focused more on the importance of cognition in risk assessment process, while paying less attention to individual emotional expression. During COVID-19 pandemic, individuals became more attentive to information about their surroundings and their own health conditions. Information from various channels can influence individual cognition and behaviors. In this circumstance, individual’s behaviors are not only influenced by cognitive risk perception, which derived from official information, but also influenced by affective risk perception that derived from surrounding environment. Consequently, in the context of a health crisis, understanding individuals’ willingness to conform from the perspective of social influence theory can better assist government in managing and maintaining social stability. In this study, risk perception was divided into affective and cognitive risk perception to more comprehensively show the impact of different risk perception paths on individual conformity behavior (Paul and Peters, 2006; Sobkow et al., 2016). Accordingly, we made the following hypotheses:

H1a: The higher the individual’s affective risk perception, the more susceptible to normative influence.

H1b: The higher the individual’s affective risk perception, the more susceptible to informative influence.

H2a: The higher the individual’s cognitive risk perception, the more susceptible to normative influence.

H2b: The higher the individual’s cognitive risk perception, the more susceptible to informative influence.

H3a: The higher the individual risk knowledge, the less likely they are to be affected by normative influence.

H3b: The higher the individual risk knowledge, the less likely they are to be affected by informative influence.

### 2.4 Conformity behavior

Conformity refers to the tendency to change one’s behavior to align with the behavior of the group (Wang et al., 2014). According to the reasonable behavior model, most human behaviors are under one’s own control, and individual behaviors are reasonable given the circumstances (Ajzen and Fishbein, 1973; Shim et al., 2001). Research has shown that consumers consider and accept the influence of other people’s information when limited information is available, and that this is an effective way to make a correct judgment. In the process of consumption, consumers can help themselves to make correct decisions using information from the outside world (Shim et al., 2001). Information structure is one of the most important factors that lead to conformity behavior. When people are uncertain about the information they have obtained and believe that the information held by others is more effective, their conformity intention will increase. Especially, if the information comes from their friends, they may exhibit more conformity behavior (Jia and Liu, 2017). In addition, conformity behavior also has social purpose. To meet the in-group’s expectations, facilitate social affiliation (Baumeister and Leary, 2017) and obtain rewards or avoid punishment (Wyer, 1966), the individual conforms to others’ opinions and behaviors. The panic experienced by the individual in an epidemic situation means that the normative influence effect will modify consumers’ behaviors after they have discussed with other people (Theodoridis and Zacharatos, 2022). When the government does not provide the latest information, rumors can emerge, which also bring psychological distress (Garfin et al., 2020). When the individual lacks information (be it because officials do not understand the information or because the information is not efficiently communicated), ambiguity can lead to more stringent assessments of the individual’s threats, resulting in greater uncertainty and chaos (Eger et al., 2021). This can result in anxiety, which may lead to conformity behavior. Consumers exchange information via public or information channels so as to modify their behavior (Garfin et al., 2020); we examined this influence of information in the context of the COVID-19 pandemic.

The stronger the normative social influence perceived by individuals, the stronger their willingness to conform (Katona et al., 2011). Public health crisis events force group members to interpret the impact of crisis events in a short period of time, and redefine the common practice through revision of the situation to form new behavioral norms and principles. Once social emergency norms have emerged, these will also exert normative pressure on individuals, encouraging them to imitate and conform, which will lead to group gathering behavior (Wang et al., 2019).

Social influence refers to the change of attitudes or behaviors of the affected people after they have accessed information from affected people. People are primarily affected by normative influence and informative influence. Normative influence refers to the normative pressure generated by the members around. The affected people will reduce this normative pressure by adopting behaviors consistent with other individuals or groups (Abrams and Hogg, 1990).

When the individual lacks information (be it because officials do not understand the information or because the information is not efficiently communicated), ambiguity can lead to more stringent assessments of the public crisis; this results in greater uncertainty and chaos, and resulting anxiety may lead to conformity behavior (Taha et al., 2014). Similarly, one study reported that when official updates were not provided about a school shooting, rumors proliferated, along with psychological distress (Jones et al., 2017). When these ambiguities are combined with an invisible threat, such as a virus, fear and worry may be exacerbated, and this could contribute to the spread of misinformation (Lv et al., 2022). Normative influence emphasizes the consistency between the affected person and the influencer. The influenced person mainly aims to maintain a closer social relationship, and exhibits a kind of value identity and behavioral compliance, whereby the informative influence of those affected will be stronger (Abrams and Hogg, 1990). In summary, we made the following hypotheses:

H4a: The greater the normative influence on the individual, the higher their conformity behavior.

H4b: The greater the informative influence on the individual, the higher their conformity behavior.

**Figure 1:**
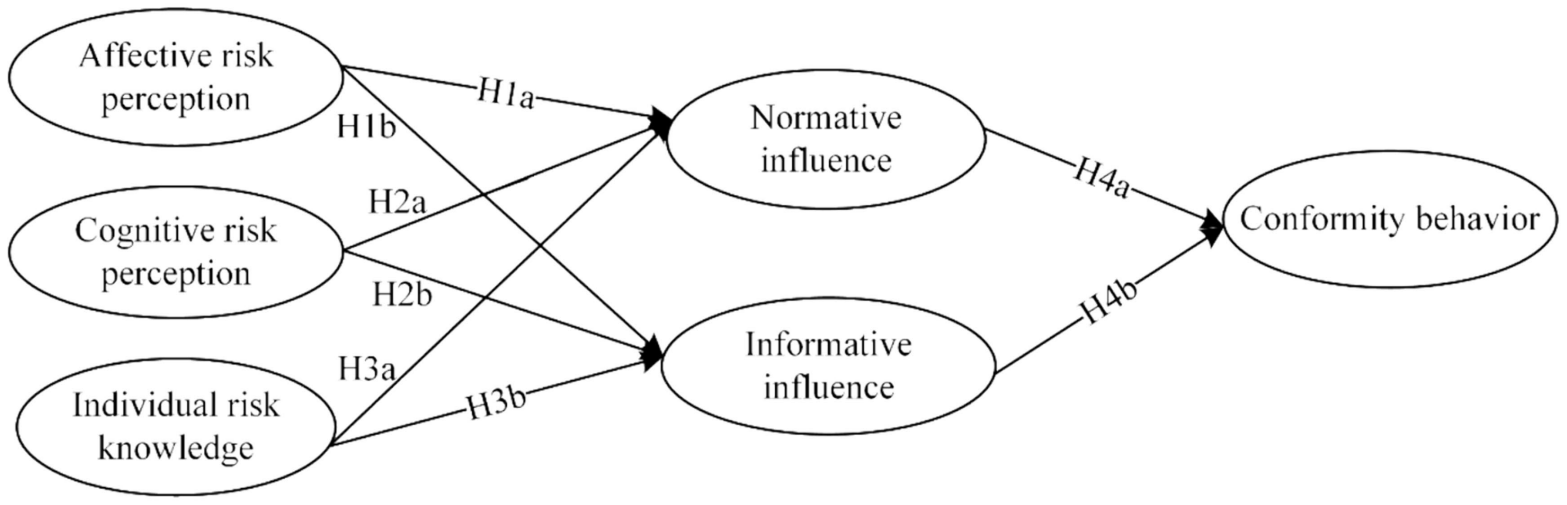
Research model.

The research model was as follows (Figure 1).

## 3 Methods

### 3.1 Data collection

We collected data using a self-administered online survey that was completed by 708 Chinese citizens from February 10 to March 10, 2020, the time at which the COVID-19 pandemic was at its worst. At that time, the threat of COVID-19 to Chinese citizens had reached its peak, as did the individual’s anxiety levels. The unique real-time data collected during the crisis represent the individual’s willingness to engage in conformity behavior and respond to risk perception, which is more convincing than retrospective reports or hypothetical scenarios.

In February, when COVID-19 was at its worst in China, there were rumors that Shuanghuanglian Oral Liquid could prevent infection, and individuals lined up overnight to buy this medicine. The respondents were asked if they had been hoarding and buying Shuanghuanglian Oral Liquid, stocking up on daily necessities, and/or transmitting various epidemic-related information in WeChat groups. All respondents were required to have one or more of the above three types of behaviors; second, restrictions were designed to ensure that each interviewee could only participate in one answer. We exclude 110 samples according on the principle of response time and attention detective questions. Finally, 598 questionnaires were collected. Our sample was 57% male and 43% female, and all detailed demographics are reported in Table 1.

**Table 1:** Sample demographic information.

Demographics	No.	% (Approximately)
Sex		
Female	257	43
Male	341	57
Healthy		
Excellent health	264	44
Fine	271	46
Some chronic diseases	35	5.8
Some cold symptoms	23	3.8
Terrible health	5	0.8
Education		
Completed Year 12 or less	22	3.7
Junior college degree	193	32.2
Undergraduate degree	201	33.6
Postgraduate degree	148	24.7
PhD	34	5.7
Age		
18–25 years old	261	43.6
26–30 years old	95	15.8
31–40 years old	155	25.9
41–50 years old	55	9.1
51–60 years old	21	3.5
61–70 years old	11	1.8

### 3.2 Measures

This study adopted measures from the existing literature and adjusted them according to the research context. Affective risk perception and cognitive risk perception were measured using eight items from Ng et al. (2018), individual risk knowledge was measured using four items adopted from Füller et al. (2008), normative influence and informative influence were measured using eight items from Bearden et al. (1989), and conformity behavior was measured using four items from Bearden et al. (1989). We used a self-administered survey, and all items were rated on a seven-point Likert scale ranging from “strongly disagree” to “strongly agree.”

### 3.3 Measurement model

We used Mplus version 8.0 and SPSS version 22.0 to analyze. A six-factor measurement model was estimated using CFA, and the model results showed an acceptable fit to the data, χ^2^(142) = 459.495 (*p* < 0.001), RMSEA = 0.061, CFI = 0.958, TLI = 0.949. All items had a factor loading that exceeded 0.7 (Hair et al., 2010), except the item “I have enough experience and knowledge to deal with the outbreak of COVID-19” in the affective cognitive risk perception measure, and “I once advised my friends to forward such messages” in the conformity behavior measure. We deleted these two items and re-estimated the measurement model, after which the model fit the data, χ^2^ (125) = 327.226.845 (*p* < 0.001), RMSEA = 0.055, CFI = 0.969, TLI = 0.962.

The factor loadings, Cronbach’s α, CR, and AVE are reported for all items in Table 2. Table 2 summarizes the AVE, Cronbach’s α, and the CR values for all constructs. The results (see Table 2) show that all constructs had a factor loading ≥0.7, a Cronbach’s α ≥ 0.7, CR ≥ 0.7, and AVE ≥ 0.5, and all were therefore deemed sufficient (Fornell and Larcker, 1981). All factor loadings exceeded 0.7 and were significant at the *p* < 0.001 level, which indicates convergent validity (Hair et al., 2010). Finally, the square root of the AVE values for each construct were higher than the correlations between all constructs (Fornell and Larcker, 1981), which supports the discriminant validity of the constructs (see Table 2).

**Table 2:** Operationalization of constructs.

Variables	Items	Factor loading	CR	AVE
Individual risk knowledge	I think I have a more comprehensive understanding of this epidemic.	0.773	0.874	0.634
	I understand professional terms such as nucleic acid detection.	0.782		
	I understand the incubation period, transmission route, and harm of the new coronavirus.	0.862		
	I understand that in this epidemic, the state provided financial support and other similar policies.	0.765		
Affective risk perception	I worry that COVID-19 will be transmitted to my family.	0.735	0.933	0.774
	COVID-19 is a serious threat to me.	0.952		
	The current situation makes my family and I very nervous.	0.928		
	I feel nervous when I think about the current epidemic.	0.900		
Cognitive risk perception	Our living habits are good, I think the chance of infection for me and my family is very low.	0.866	0.920	0.793
	Compared with others, my family and I are less likely to be infected.	0.955		
	I think the possibility of me being infected by COVID-19 is very low.	0.847		
Informative influence	Some public accounts on the Internet or Weibo I follow are reposting this information.	0.747	0.859	0.672
	The frequency of reposting the information around is of great reference significance to whether I want to repost it.	0.899		
	If many people around are reposting it, it is inferred that I should repost this information.	0.806		
Normative influence	As far as I know, my friends have also reposted these COVID-19 related news/have purchased spare medicinal materials/daily necessities.	0.736	0.891	0.674
	I reposted these messages when I saw that my friends were reposting them/had purchased spare medicinal materials/daily necessities.	0.824		
	My friends also support me to forward this type of epidemic-related information/purchase spare medicinal materials/daily necessities.	0.903		
	As far as I know, most people once wanted to forward news related to this type of epidemic/purchase spare medicinal materials/daily necessities.	0.811		
Conformity behavior	I will continue to forward this information/purchase spare medicinal materials or daily necessities.	0.732	0.804	0.578
	I have forwarded this information/hoarded these medicines or daily necessities.	0.731		
	I once advised my relatives and friends to forward this information/hoard these medicines or daily necessities.	0.815		

### 3.4 Structural model

**Figure 2:**
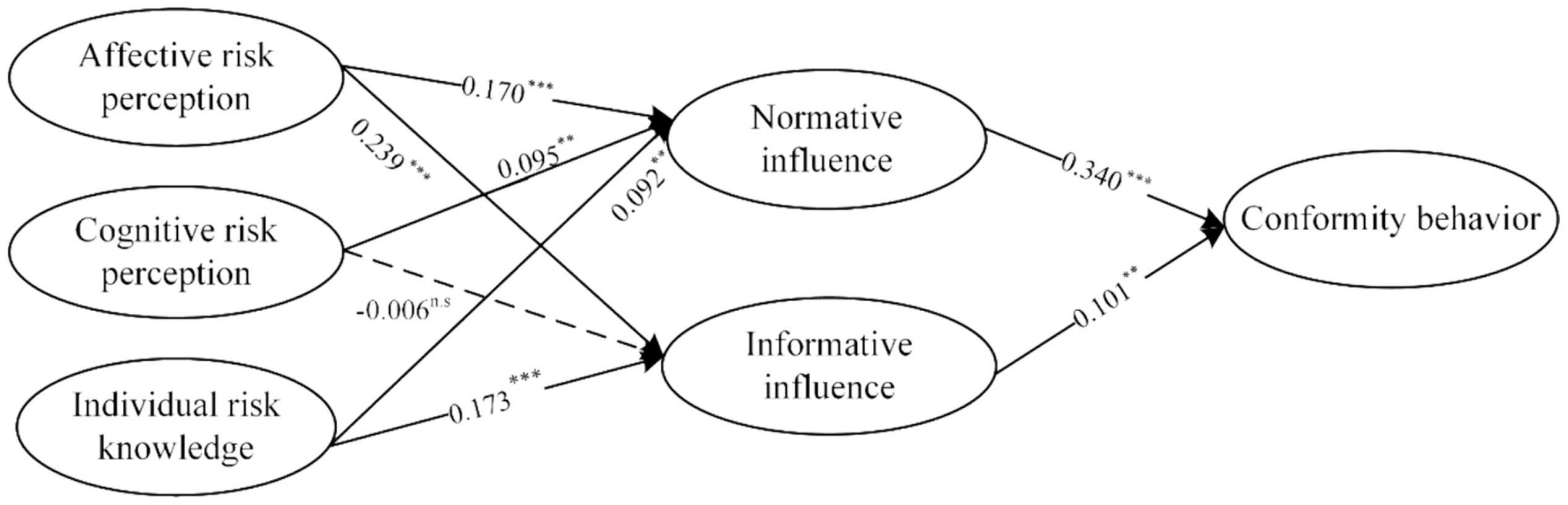
SEM results. ^**^p < 0.05; ^***^p < 0.001.

This study used the SEM method to test all hypotheses, and Mplus version 8.0 was used. This structural model fit the data adequately, χ^2^ (178) = 430.845 (*p* < 0.001), RMSEA = 0.049, CFI = 0.968, TLI = 0.962. Figure 2 shows the standardized regression weights of the causal paths in the model.

Affective risk perception (β = 0.170, *p* < 0.005), cognitive risk perception (β = 0.095, *p* < 0.005), and individual risk knowledge (β = 0.092, *p* < 0.05) had a significant positive impact on normative influence, thus supporting H1a and H2a. H3a was not supported. Compared with cognitive risk perception and individual risk knowledge, affective risk perception has greater impact on normative influence. Affective risk perception (β = 0.239, *p* < 0.005) and individual risk knowledge (β = 0.092, *p* < 0.05) had a significant positive effect on informative influence, thus supporting H1b. H3b was not supported. Cognitive risk perception had no significant effect on informative influence, and H2b was therefore not supported. By comparing the influence of different independent variables on mediating variables, we have found that affective risk perception has greater impact on normative influence and informative influence compared with cognitive risk perception and individual risk knowledge. If the independent variable is fixed, affective risk perception and individual risk knowledge effect informative influence more. And cognitive risk perception is significant only if the mediating variable is normative influence. Informative influence (β = 0.101, *p* < 0.005) and normative influence (β = 0.340, *p* < 0.005) had a significant positive effect on conformity behavior, thus supporting H4a and H4b. The normative influence has greater impact on conformity behavior. Then, we calculated the mediating effect size of the two mediating variables separately. The mediating effect of normative influence (indirect effect = 0.121) was greater than that of informative influence (indirect effect = 0.042).

## 4 Conclusion

### 4.1 Discussion

This study examined the individual’s risk perception and the impact of conformity behavior during the COVID-19 mass health crisis in China using an online survey. It has discussed the global significance issue that how to manage public behaviors during health crises and provided insights for public health strategies of government. This article expands upon the rational behavior model by incorporating individual risk knowledge into the research model, and emphasizing that risk perception is the result of the interaction between affective and cognitive.

We found that the more the individual knows about risk that is, a higher level of risk knowledge, the more extreme the conformity behavior is. This is a very interesting result, but shows that the more the individual know about risk during a public health crisis, the more nervous they will be; that said, some information-based conformity behavior and normative conformity behavior will also occur. This is similar to the conclusions of previous studies showing that the exposure to information during the new crown epidemic is positively correlated with public sentiment (Garfin et al., 2020). The greater the normative and informative influence, the higher conformity behavior. The higher the affective risk perception, the higher the normative and informative influence, and the higher the conformity behavior. While higher cognitive risk perception makes higher normative influence that induces higher conformity behavior. But cognitive risk perception has no impact on informative influence. As a result, we infer that cognitive risk perception requires individuals to invest certain cognitive resources in thinking and analysis. In this case, when individual receives unconvincing information, they will analyze the information source and content. It will not be easy to believe. Therefore, cognitive risk perception has no impact on informative influence.

Therefore, one simple and effective crisis management policy could be to increase the transparency of public crisis events and increase publicity channels so that the public has more knowledge about health crisis events, which could in turn reduce individual conformity behavior. After the government discloses the health crisis incident via formal channels, the individual is more likely to trust the government and consciously reduce dissemination of untrue information. This would mean that the government could devote more energy to other management tasks.

### 4.2 Theoretical implications

These unique real-time data collected during the crisis represent the individual’s willingness to engage in conformity behavior and characterizes their response to risk perception, which is more convincing than retrospective reports or hypothetical scenarios. The present results also expand the theoretical model of public health information dissemination channel selection. Individual behavior in crisis events has been the focus of several researchers, and this paper extends the rational behavior model. In mass health crises, adding the antecedent variables that affect informative and normative influence of the rational behavior model results in a model that has a good explanatory power (Bearden et al., 1989; Wang et al., 2019).

We explored antecedent variables that affect the informative and normative influence in the context of the COVID-19 health crisis. We further examined the mechanism underlying the effect of risk perception on individual conformity behavior and consider risk perception in circumstance of health crises from two perspectives, i.e., cognitive risk perception and affective risk perception. It not only contributes existing studies pertaining risk perception and conformity behavior, but also deepens our understanding of social influence theory (Bearden et al., 1989). Previous studies have pointed out that the individual’s various decision-making behaviors are affected by other related groups, but have rarely explored how social influence results in conformity behavior that specifically affects the public (Wang et al., 2019). In this study, we explain conformity behavior from perspective of social influence theory. It shows that in circumstance of health crises, either official information or ideas from opinion leaders can make public impact by normative influence on one hand. On the other hand, the uncertainty derived from anxiety in crises make public impact more by informative influence. Both these induce the conformity behavior. This article combined the individual’s risk perception with the social influence theory, and found that the individual’s conformity behavior is affected by normative and informative influence. The individual’s perceived affective risk and cognitive risk both affected the normative influence. Compared with cognitive risk perception, affective risk perception has a greater impact on informative influence. Unlike previous studies, we found that the individual’s risk knowledge increases the strength informative and normative influence (Füller et al., 2008). Namely, we found that when the individual has more knowledge about the public crisis, there is a greater normative and informative influence.

### 4.3 Managerial implications

To effectively respond to the COVID-19 pandemic, governments should take effective measures to reduce individual conformity behavior. When the individual’s cognitive risk perception is increased, panic will be generated, thereby increasing the possibility of being affected by information influence and normative influence. The generation of individual cognitive risk perception mainly comes from the untimely release of relevant information, which augments the individual’s risk perception. Effective government measures and timely information disclosure could reduce the individual’s affective risk perception. Specifically, agencies should proactively publish information as quickly as possible to actively guide the individual’s attention. For example, during COVID-19 pandemic, Chinese official website publishes the latest epidemic situation and response policies, which can be easily accessed by public. In managing public opinion during public crises, authenticity and accuracy are key qualities that the government should adopt to better guide the public.

A higher affective risk perception of the public is dependent on the individual’s lack of understanding of the mass health crisis; this results in the individual’s increased awareness of risk, which leads to social panic, fear, negative emotions, and a lack of a sense of security, and these will be affected by normative influence, which leads to conformity behavior. Thus, the government should establish a strong and powerful image when public health crises begin, as well as the ability and confidence to control the crisis. During COVID-19 pandemic, the strictest epidemic prevention measures have been taken by Chinese government. It had showcased the determination and ability to fight the epidemic and established a strong normative image for public through carrying out large-scale medical treatment and claiming to prioritize people’s life safety and physical health. These management measures should help reduce the public’s affective risk perception.

In the critical period of public health crisis prevention and control, the public are eager to obtain relevant information, largely because not much is known about COVID-19. Diversified management methods should be included in the management process of public health crises. These could include actively using the power of official authoritative media and government at all levels for effective propaganda and public opinion guidance to increase the public’s access to relevant knowledge and reduce the possibility of being affected by informative influence.

For example, during the handling of the “Purchase Shuanghuanglian oral liquid” incident, mainstream media made full use of resources to gradually persuade the public to refrain from blind obedience through, for example, expert explanations and professional analysis, thereby avoiding larger-scale conformity buying; this is similar to the handling of SARS in 2003. Furthermore, our results could stimulate public opinion forces and non-governmental communication platforms to supervise and report information about public health crises, which would not only enhance the transparency and public satisfaction of public opinion, but also receive more support from the public.

Given that the Internet can affect a wide range of conformity behavior, we recommended that the display of news comment information on websites are standardized. According to the spiral theory of silence, in the public opinion field, a small number of people with different opinions will give up expressing their opinions because most people hold different opinions from themselves. Over time, this will lead to a single dominating opinion in the media. To avoid the spiral effect of silence and allow different views to be expressed on the network, we should present all the various viewpoints and interpret information according to these. In doing so, an information balance will be achieved, and the self-purification ability of the network will work (Garfin et al., 2020).

### 4.4 Limitations and future research directions

This study collected data during the most serious period of the public health crisis in China. Firstly, conformity behavior are influenced by country and cultures. Further supplementation of cross-cultural contextual data is needed to test whether the conclusion still holds true in other cultural contexts, because China is the country with a collectivist tradition. Secondly, ignoring control variables is another limitation of our study. Thirdly, we collected real-time data online. In fact, the impact of health crises varies in different region. Besides, social influence is not only derived from risk perception and individual knowledge. The main results of this study cannot exclude interference from other factors. In the future research, we may consider experiment approach to control these influence.

## Data availability statement

The raw data supporting the conclusions of this article will be made available by the authors, without undue reservation.

## Author contributions

YuY: Conceptualization, Project administration, Writing – original draft. SL: Investigation, Visualization, Writing – original draft. GC: Formal analysis, Methodology, Writing – review & editing. YaY: Funding acquisition, Software, Supervision, Writing – review & editing. JY: Investigation, Writing – original draft.
